# Tumors of the Mouth

**Published:** 1892-06

**Authors:** J. Taff


### Tumors of the Mouth.

BY J. TAFF, D. D. S.

Read before tho 7th District Dental Society of Ohio, May 17, 1892.

The mouth is a part of the human organism that is subject to a variety of diseased conditions, all of which are likely to come under the observation of the dentist. These affections may be of the most simple variety of inflammation, accompanied by suppuration and mild forms of ulceration, but they are oftentimes found in more serious or violent forms.

Tumors of great variety are likely to occur in the mouth, perhaps, the more simple form of these is that usually denominated "epulis," which term simply means, upon or springing from the gums. These more frequently are simply benign growths varying in consistence from merely a fungoid form to that which may be very firm and resistant. They sometimes consist of simply one large lobe or mass; in others they are lobulated, consisting of two or more miniature lobes, either but little, and in some cases, very markedly fissured. They are sometimes of very rapid growth, and in others quite slow; months, even years, sometimes being required for any considerable development; in other instances they spring into considerable prominence within a few weeks time. They usually start from the margins of the gums, between two or more teeth, sometimes enveloping the free margin along two or more teeth. Their structure varies very greatly, sometimes it consists simply of a fungoid mass, very freely ramified by blood vessels, and still very poorly organized. In others to be less vascular more dense, of a myeloid character with little or no apparent organization. In the growths of medium, or less rapid formation, they are oftentimes found of fibrous character throughout, a good degree of organization being effected. These are more dense and resistant than those already mentioned. These tumors are well nigh, if not altogether, cartilagenous, having a very small amount of blood supply. None of these tumors cause persistent pain, and very seldom any appreciable soreness.

The attachment of the more vascular varieties will usually be by a small base, sometimes, often little more than a thread-like attachment, through which an artery and vein pass. Others will have larger attachments or broader base, and extend deeper into the structure, and be more likely to involve the surrounding and deeper structures; in such the diameter of the attachment may be larger than the tumor; elsewhere this variety of tumors may grow to a large size, and not in any serious extent involve the adjacent tissues. It is very seldom indeed that this simple form of tumors become attached to the bony tissues; such occurrence would only be likely to take place where the growth was of long standing and persistent development,

The circulation through these tumors is oftentimes so nearly normal as to cause but little, if any, change in the color of the mucous membrane enveloping them. In all cases, however, where there is greatly increased vascularity, there will be heightening of color, and sometimes a purple hue is presented; this depending upon the sluggish character of the circulation. In a well nourished and vigorous constitution, these growths will be less rapid and less likely to encroach upon structures much beneath the surface. In studying and dealing with these growths, it should not be forgotten that in favorable circumstances with a low grade of vitality and a strumous cachenia that a more or less malignant character may be assumed; they, like many other things, will proliferate and grow in a favorable soil. The lower the tone of the system, the less perfect the nutrition, and especially if there is present some special vitiation, the greater is the liability to such growths.

It is not always an easy matter to determine the origin or cause; in some cases there is no difficulty in this respect, in others the origin is very obscure. They are sometimes found upon the gum beneath which there is an accumulation of salivary calculus, in others an accumulation of other offensive material. I do not remember to have ever seen a case of epulis occurring because of pyorrhœa alveolaris, but they do occur upon diseased gums, induced by roots of teeth, oftentimes the merest remnant will be sufficient to do this. In nearly all cases where these tumors exsist upon the gum in connection with sound teeth, there will be somewhat of separation of the gum from the neck of the tooth, but usually little or no suppuration.

### TREATMENT OF THESE TUMORS.

Proceeding with special interference to treatment of these tumors, the true character in every case should be thoroughly understood. If of the more simple forms with small or comparatively small basal attachment, a prompt removal may be made with the scissors or knife, seizing the growth with a pair of toothed forceps make a clean cut from the bottom, remove the whole in one mass, there will always be some hemorrhage; in the more vascular varieties, this may seem in some instances to be almost alarming; blood will spurt with considerable force from the vessels, in some cases, exhibiting an arterial pulsation, in other cases, though flowing very freely, will be continuous or without the intermittent character. This, however, is usually easily arrested by the use of proper hæmostatics and with compression. If the base is larger, and the tumor seemingly very vascular, a very effective and perhaps less objectional way to remove it is by ligation, which results simply in strangulation; when sloughing will ensue and the tumor be removed. The principal objection to this method of procedure, however, is that all the tissue involved will not be so certainly removed as would be in the use of the knife or scissors; but with the ligature, there is very little liability of hemorrhage, unless there is a hemorrhagic tendency. Such a predisposition should always be ascertained before proceeding with even the most trivial operation of this sort.

These tumors having the large attachment give the greater difficulty in their removal, and special care should be exercised in such cases, for they are more likely to involve the tissues more deeply.

Another method of treatment of certain forms of these growths, is by injection of some preparation that will either coagulate the contents of the tumor, and so interfere with the circulation, that its support and growth cannot be longer maintained, or by some material that will destroy the nutrient property of the plasm in the growth. Whenever in these structures the nutrient supply is cut off or markedly interfered with, there will not only be an arrest of the growth, but nature at once sets up a process for eradication. A solution of carbolic acid and iodine, the strength of which should be determined by the recognized conditions present, may be used in many cases with very good results. A solution of creosote and tannic acid has been employed by some, but it perhaps possesses no advantage over the preparation first mentioned. Electrolysis may be employed in some cases, with prompt and successful results. In large vascular tumors of this variety, with sluggish circulation and increased color, this method may be employed with very decided benefit; this process is accomplished by the use of the ordinary battery, connected with the electrolytic needles; these passed into the tumor, but not brought in contact, and the current being made to pass through from three to eight minutes. This exercises a destructive influence upon the nutrient material, and the mass will slough away.

---

A Doctor in Bootle, England, has the following printed on his prescription blanks: Gratefulness of the patient is part of his disease, is most prominent when the fever is highest, lessens during convalescence, and disappears as health is re-established. Hence, prescriptions only for cash.—*Memphis Medical Monthly.*

